# Dr. Samuel Denton

**Published:** 1860-11

**Authors:** 


DR. SAMUEL DENTON, Professor of Medicine in the University of Michigan, died at Ann Arbor, on the seventeenth of August.

